# Discourses Delivered by Appointment, before the Cincinnati Medical Library Association, January 9th and 10th, 1852

**Published:** 1852-04

**Authors:** 


### REVIEWS.

---

Art. IV.—*Discourses Delivered by Appointment, before the Cincinnati Medical Library Association,* January 9th and 10th, 1852. By Daniel Drake, M. D. Moore & Anderson: Cincinnati: 1852.

Belonging as medicine does to the class of the natural sciences, which rest upon observation and experience, it is hardly to be hoped that it can ever be carried to a point where it may be pronounced perfect and complete. As long as human judgment is fallible it must remain imperfect; as long as there are active, sagacious minds to observe, it must continue to be progressive and subject to change. It is far from being at this time what it was at the beginning of this century, and, we may hope, will be at the end of the century far in advance of its present position. In no two periods of its extended history has it exhibited precisely the same aspect, nor for a long time to come is it likely to be less mutable than it has been in the ages past. Progress implies change, as growth in the animal body involves metamorphosis and decay, and the more rapid the march of science, the more frequent must be its changes. From Hippocrates to Galen, from Galen to Boerhaave, medicine advanced slowly, and its revolutions succeeded each other at long intervals. Its progress during those ages as compared with the rate at which it is advancing now, is what the coasting expeditions of the Romans were to the steam navigation of our day. If the sage of Cos had been permitted to return to the scene of his labors after the lapse of a few centuries, he would have noted some changes in the doctrines which he taught, some additions to the healing art, some extension in medical science; but to his great mind the improvements made by generation after generation of his successors would not have appeared otherwise than contemptible. If Boerhaave, Haller, or Cullen could revisit the cities of which in their day they were the great medical luminaries, they would find it necessary to commence the study of their profession anew. Nay, those who quitted their studies only a few years ago, find themselves already quite behind the times. So many old opinions have grown obsolete, and so many discoveries have been made, that renewed study is felt to be indispensable for those who would keep pace with the march of their profession.

Journalism is at once the result of this improved condition of medical science, and an important contributor to its advancement. Dr. Drake, in his second discourse, which we propose now to notice at some length, gives an interesting history of the origin of medical journals, and discusses very ably their influence upon the profession. They belong to modern times, and have multiplied with the increasing activity of medical research. At first, they consisted chiefly of the papers read before medical societies, and were published at long intervals.

The first of these was begun in the year 1722, under the title, *Acta Medicorum Berolinensium*, in Berlin, Prussia. I have not seen it, but believe it was annual. Nine years afterward, A. D. 1731, another series of yearly volumes was commenced by a scoiety in Edinburgh, under the title of *Medical Essays and Observations*, and continued to six volumes. Each was divided into four parts: first, Weather and Disease in Edinburgh; second, Epidemics of the different Seasons there and elsewhere; third, Original Essays and Papers; fourth, Explanatory Diagrams and Figures; fith, Discoveries and Improvements in Medicine. Here we have substantially the plan of the most popular periodicals of the present day, from which it differed, in being an *annual* instead of a quarterly or monthly. This first periodical of our own language is in the Library of the University of Louisville. In 1753, a second German work was commenced at Leipsic, under the the title of *Commentarii de Rebus in Scientia Naturali et Medicina gestis.* In 1754, Vandermonde, of Paris, began the publication of his *Recueil Periodique d' Observations de Medicine, de Chirurgie, et Pharmacie*, which, as far as my means of research can carry me, was the first medical periodical of France. I do not know at what intervals it was published; but the title indicates that it was to be published at stated periods. Five years afterward, 1759, a second was started under the title of *Journal de Medicine.* The Edinburgh annual of 1731, which I have just named, proved to be the prototype of a quarterly magazine, in the same city, which, in plan and objects, was still more identical with the journals of the present day. It was projected, like the other, by a society, of which Dr. Andrew Duncan was secretary. The first number, bearing the title of *Medical and Philosophical Commentaries*, was issued in the month of January, 1773, and it was kept up by that distinguished physician for twenty years, when *of Medicine.* Each number was divided into four heads—he merged it in an annual volume, under the title of *Annals* first, an account of books in medicine and those branches of philosophy most intimately connected with it. Second, Medical Cases and Observations. Third, Medical News. Fourth, List of New Medical publications. Both these works are, also, in the University of Louisville. As yet London and Dublin had issued no journals; and we may take this, as the first medical *quarterly* in our language, beginning 79 years ago. The Edinburgh school was at that time in high relative distinction; and to its quickening influence, we should, I suppose, ascribe this important movement, in advance of any in the sister cities of the United Kingdom.

Dr. Drake proceeds then to offer some philosophical reflections on the cause of the tardy appearance of medical journalism, and on its influence upon the profession. The extract is a long one, but we are sure it will be read with interest.

Such was the origin of medicalcal periodi literature in Germany, France, and Great Britain, and we see that even its annual form does not date back more than 120 or 130 years; the first half of which period was but a protracted infancy. Yet the art of printing had, then, been invented more than 250 years, and a learned periodical of general literature and science, entitled the *Journal des Savans*, had been commenced in France nearly a century before. When we contrast the late and tardy development of this branch of our literature, with its progress and character since the commencement of the present century, the mind turns inquiringly after the causes of so great a difference. We may readily admit, that many of these are not peculiar to our profession; for *all* periodical literature has experienced an impulse, which might well fix the attention of the philosophical historian. Without the slightest pretension to that character, I may venture to throw out a few suggestions.

As long as physicians and other philosophers, made it their chief and cherished occupation, to pore over the writings of the ancients—Greek, Roman and Saracenic—their labors consisted largely in writing notes, commentaries, prefaces, and appendices to the books, the study of which they preferred to the study of nature. For the publication of such servile lucubrations, a periodical press was not required, and of course not established. To Bacon, the nations which speak the language of that extraordinary man, (if not the whole of Europe,) were indebted for at least the beginning of their emancipation from the shackles, which the dark ages had fixed upon them. Men began to inquire into nature as she appeared to them, and correspondingly ceased to inquire how nature had appeared to *Hippocrates*, *Aristotle*, *Galen* or *Pliny*, two thousand years before; but the results of *such* inquiry could not be recorded on the margins or blank leaves of the venerable tomes handed down from the times of those great men. Thus, *original* publications became necessary; but they did not at first assume the periodical character. The march of this reformation, or, rather, of the liberation from intellectual bondage, was onward, but not rapid; for under a traditional veneration for the *fathers*, men still sought to reconcile their own observations and conclusions with those of antiquity. Even those great observers, Sydenham, and more especially Boerhaave, made frequent reference to Hippocrates, Celsus and Galen; yet both presented so much originality, as to bring many of *their* cotemporaries and successors into a state of admiring dependence, and secure for themselves commentators as patient and prolix as any who had before honored the *first* fathers; the idolatry continued, though in a diminishing degree, but the idols were changed. In our own country this kind of reverence has not yet become extinct; but, in a modified and harmless form displays itself in the introductions and annotations with which so many of us enlarge or illustrate the writings of our European cotemporaries.

Since the emancipation of mind from a slavish devotion to the mind which went before it, periodical literature, in every department of knowledge, has waxed greater and greater in volume and originality, until not only in medicine, but all the sciences, it has become a paramount element. It is not only the first to present all discoveries and inventions, but actually supplies to the masses almost every thing they read. The review of a book they prefer to the book itself. If the reviewer condemn it, they think it unworthy of being read; if he praise it, they may purchase, but will seldom study it. This gives to journalism in every department of human knowledge a mighty power, which, to its own glory, it has created for itself. Its own hands have placed the crown upon its brow, and given it a dominion over the intellect and feelings of the world, which it will never abdicate, and from which it can never be cast down. Even at the present time, when we cannot believe its influence fully developed, its power sets every estimate at defiance, and its extinction would arrest the progress of civilization. But its own growth has been signally promoted by the civilization of which it is *pars maxima*. Thus, the ways and means of internal and external personal and national intercourse and conveyance, which constitute distinguishing features of modern society, so obviously and powerfully favor the progress of every form and kind of journalism, that in our inquiry after the causes of extension, magnitude and power, in the middle of the nineteenth century, compared with that of the eighteenth, we are required to ascribe a large influence to agencies entirely material, and connected only with publication and distribution.

This extended journalism, the effect of one revolution in medical science, is becoming the cause of another. Before it was brought into existence, the inventions and discoveries in each country found their way but tardily among its own physicians, and many of them never passed its boundaries. Thus, each nation acquired an idiosyncracy, which would have been far less strongly marked, if its opinions, prejudices and traditions, had been brought into contact with those of other nations. National peculiarities in pathology and practice, especially the latter, will always exist; but they *should* be no greater than logically flow from the peculiar, physical and moral conditions of each country, after they have been compared with and neutralized by every other. Now, it is one of the missions of journalism to forward this comparison, and by augmenting the *common*, to diminish the *peculiar*.

The national interchange and extension of improvements in medicine, can be effected in three modes only. *First.*—By the reciprocal attendance of students and visiting physicians, on the Universities and Hospitals of different nations. But this is personal, and wholly insufficient for the instruction or information of the majority, who are never taught out of their native land. *Second.*—By the importation or re-publication of monographs or systematic treatises. This, in some cases may be sufficient, as, for example, the physicians of the United States may, in this mode, acquire a knowledge of English, Scotch, and Irish medicine; but those older countries are not likely to import or re-publish many of our works. And when we turn from those who speak and write the English language, to the continent of Europe, we find the difficulties in this method of interchange greatly increased, for we there see medicine cultivated in the French, German, Italian, Spanish, Polish, Russian, Swedish, Latin, and other languages, and a necessity for translators to prepare the works of one nation for re-publication in another. This imposes a severe restriction on the dissemination of knowledge, because many works containing matter which might be new to the physicians of other countries, at the same time include so much that is common to the whole, as to render it unsafe for translators and publishers to engage in their dissemination. Thus we are driven on the *third* method, or that of the periodical press, which we have already discussed.

By the development which this method has recently acquired, and which is still going forward, every nation is, or may be put into cheap and early possession of all the important discoveries, inventions, improvements, and new conclusions of every other. Thus, each is correcting its own errors, and extending the knowledge and influence of its own truths by the aid of all the rest. The discovery of a new remedy in Guy's Hospital is telegraphed to Paris, and prescribed the next day in the Hotel Dieu; it proves ineffective, the patient dies, a new form of pathological lesion is found, a Paris hebdomadal carries the record back to London, and in a single month the periodical press makes it known throughout Great Britain and Ireland. Edinburgh develops a new truth in hygiene or surgery, which her periodicals transmit to Berlin, whence, by the same instrumentality, it is spread over Germany. By the same (figurative) speaking trumpet, the physicians of the Mediterranean instruct those of the Baltic, and are instructed in turn; London, St. Petersburgh, and Calcutta compare their experience, and Edinburgh, Pisa, Vienna, and Paris, in the same language, proclaim their scientific discoveries to the world.

But to look at this subject in its full magnitude, we must now contemplate it in our own country. Every packet brings to our shores the periodical issues of Great Britain and Ireland, France, Germany, and Italy; and, on returning carries to the old the humbler contributions of the young world. That which once required years to reach us, now comes in as many weeks; and that which was formerly seen by hundreds only is now read by thousands. The discoveries and practical improvements in physic and surgery of our elder and abler brethren of Europe, are no longer thought of, as revelations, which may or may not reach us, till our eyes are too dim to read them, our hands too old and stiff to adopt new modes of operating, but they come fresh and warm, and full of life-juice, and, like young and succulent buds, readily take root in the stock of our existing knowledge. Even the village surgeon now cuts according to the newest fashion of some great transatlantic operator; and when two country physicians meet for consultation in the log cabin of a backwoodsman, they discuss the propriety of a practice, which, but thirty days before, had been *professed by a Professor* in one of the ancient universities of Europe. Thus, the voice of the Thames, the Danube, or the Seine, is heard, repeated, and approved or condemned, by that of the Ohio or the Missouri; and this brings us to the special consideration of our own periodical literature, without the aid of which, this phenomenon of unlimited and rapid diffusion could not have appeared.

After this follows a history of the origin of medical journals in this country, which we append:

Medical Journalism in the new world did not begin as a mere reprint or humble imitation of that sent us from the old; very little of which had indeed reached us, at the period when our own commenced, for, in truth, the old had then but little to send. So limited, indeed, had been the patronage of Great Britain and Ireland, that after publishing a quarterly for twenty years, Dr. Duncan, in 1795, took the back step of returning to an annual volume. It was at this very moment of British retrogression, that America was preparing to make her appearance on the theatre of medical journalism. New York has the honor of having led in this enterprise; and three native physicians, who had never visited Europe, Dr. Samuel L. Mitchell of that city, Dr. Elihu H. Smith of Connecticut, and Dr. Edward Miller of Delaware, have the distinction of being its projectors and conductors. On the 26th of July, 1797, the founders of the periodical literature, sent forth the first number of the *New York Medical Repository*, which was continued in quarterly issues till twenty volumes, (and perhaps more,) were published. Before the second was completed, the brightest medical genius of the editorial triumvirate, Dr. Smith, fell a victim to yellow fever, then epidemic in New York, but the others continued the work without interruption. During the publication of the fifteenth volume, Dr. Miller also died, and it was afterwards conducted by Dr. Mitchell, Dr Felix Pascalis, and Dr. Samuel Ackerly. A copy of this work is in our library, of which it very properly constitutes the corner stone.*

The second American periodical was commenced in Philadelphia, A. D. 1803, by Professor Benjamin Smith Barton, under the title of the *Philadelphia Medical and Physical Journal.* It was devoted to zoology and botany, not less than medicine, and partly on that account, perhaps, expired, at the end of the third volume. It was, however, immediately followed by the *Medical Museum,* which was continued for six years, edited by Dr. John Redman Coxe, now the oldest living editor in the United States. Copies of both these early periodicals are in our rooms.

A few years afterwards Baltimore entered the list. In 1809, Dr. Tobias Watkins commenced and completed one volume of the *Baltimore Medical and Physical Recorder;* and, in 1811, Dr. Nathaniel Potter brought out another under the title of the *Baltimore Medical and Philos. Lyceum.* Both of which are on our shelves.

At a period still later, Boston made her appearance in the field, by the publication, January, 1812, of the first number of the *New England Journal of Medicine and Surgery*, conducted by a number of physicians. It was published quarterly. This, also, is in our collection.

Thus, in the course of fifteen years, from 1797 to 1812, medical journalism was implanted in all the great cities of our sea-board. True to its *American* connection with medical schools, it has, however, flourished most in Philadelphia, which, for half a century, has taken the lead in medical education. Within that period, many journals have been started in that city and elsewhere eastof the mountains. Some of which soon expired, others were absorbed by pre-existing publications, and others have held on their way.

Before leaving the states of the sea-board for those of the interior, I must refer to the origin of a journal devo-

*As Dr. Duncan had reconverted his quarterly into a yearly publica tion, in 1795, there was not, I believe, in the English language, a med ical quarterly or monthly, at the time the New York Repository was first issued. This had led some persons into the error, that it was the first ever published in our language. For a while it was the only one.

ted to the sciences auxiliary to medicine. Here, again, New York attempted the lead, but failed. In the year 1810, Dr. Archibald Bruce commenced, in that city, a Journal of Mineralogy, but it ceased never to be revived, at or before the end of the first volume; after which, as far as I know, nothing further was attempted for the next seven or eight years.

In the year 1817, Dr. Benjamin Silliman, Professor in Yale College, projected a periodical to be devoted to *Physics* and *Natural History* generally. At New Haven, in the month of July, 1818, the first number appeared, under the title of *American Journal of Science and Art*, and it has been continued, bi-monthly, ever since. For the first thirty-three volumes, he was the sole editor; then his son, B. Silliman, Jun., now Professor of Chemistry in the Medical Department of the University of Louisville, became his associate; and, on the commencement of the fifty-first volume, Professor J. D. Dana, of Yale College, was added to the number. This journal, running through a third of a century, contains a vast number of papers, intimately connected with medicine, and, therefore, richly deserves a notice among our own first periodicals. It has gone, hand in hand. with the American Journal of the Medical Sciences, in making us known to the *savans* of Europe—a proud, self-erected monument, to the unconquerable perseverance of its distinguished founder.

But my object being to indicate the *beginnings* of our periodical literature, I shall not attempt further to follow the history of eastern enterprise, but will now proceed to speak of those beginnings in our own interior valley.

Lexington and Cincinnati were for many years the only seats of medical learning in the interior, and for many years afforded the only medical journals; "but latterly," as remarked by Dr. Drake, "the number has become great." In all the country there are now thirty-two published; of which there are seven in New York, five in Pennsylvania, two in Louisiana, two in Kentucky, and three in Ohio, and in New Hampshire, Massachusetts, Maryland, Virginia, New Jersey, South Carolina, Georgia, Illinois, Missouri, and Iowa, each one. In Canada there are two. Dr. Drake compares the increase in the number of journals with the increase of population, and finds that the rates have been very different. Thus—

In 1850 the population was only three and three-fourth times above what it was in 1800; but the number of journals was thirty times as great; thus the increase of the latter was eight times as great as the former. When we compare our population and journals with those of Great Britain, and Ireland, the result is striking. Prof. Lawson has informed me that the United Kingdom has but fifteen journals, which gives one for every 1,830,000 inhabitants; while in this country, there is one for every 715,000. I can not extend the comparison to the continent of Europe, but feel quite certain that the number of our journals is greater for the number of people, than in any other nation of the earth.

What is the cause of this remarkable fecundity? Is it because we are greater readers and more industrious writers than the people of any other country? Dr. Drake shall be allowed to speak to this question. He says—

This remarkable multiplication should arrest our attention, both as to its causes and effects; and first of the former. Does it indicate either a great expansion of our periodical literature? an immense number of readerrs? or a multitude of writers? I must answer the whole of these questions in the negative. A long continued connection with the Western Journal, and much intercourse with publishers and Editors, on both sides of the mountains, have convinced me, that a large proportiou of our journals have subscription lists, which compared with those of Great Britain, are (as I suppose) exceedingly limited. Very few of the whole, afford any profit to their publishers, and still fewer any compensation to their Editors; while scarcely one pays anything to contributors.— There is no mistaking the import of these facts, which point directly to the conclusion, that we can not estimate the number of readers, by the number of our journals. It is not, then, strictly speaking, a demand, which has carried their number up to thirty, instead of thirteen, as

4

the British proportion would give, but some other cause or causes.

The first which occurs to my mind, lies imbedded in our national character, and was developed there by our free institutions. The daily, weekly and monthly journals of the United States are more diversified and numerous than those of all the world beside. Henceforth and forever the political philosopher may hold, that, *cæteris paribus*, their number will be directly as the freedom—inversely as the absolutism of government; and need ask no other exponent—seek for no other sign—would find no surer if he did—than the proportion of political and social periodicals, compared with the population of a country. Journalism, then, is an American—a republican—propensity; and it would be remarkable, indeed, if it did not display itself beyond the limits of business, politics, religion, popular education, and polite literature.

But there are causes in the medical profession which quicken and sustain this propensity in great activity. Our Union consists of sovereign States, in each of which there is an *esprit du corps*, and a metropolitan center; each has newspapers devoted to its own interests, and it would be strange, if it did not aspire to other and higher publications. England, Scotland, and Ireland manifest this individuality, and have more journals than if they were one people, like the French; but the States of our Union are ten times as numerous as those of the United Kingdom, and of course our medical journals are multiplied correspondingly. But the indirect influence of our federal system, is still greater than the direct. Each state is emulous of every other; an aspiration of eminent advantage on the whole, but sometimes injurious to particular interests. Under the influence of this emulation, we find our State legislature at all times ready to grant charters for medical education; and as American physicians, in a strange delusion, theoretically attach greater pleasure and dignity to the teaching, than the practice of medicine, they are ever applying for chartered privileges. Thus we already have more medical schools than we have states or journals; and are likely to have the number of both increased every year. Now the greater part of the latter, from the beginning, have been projected by those who belonged to medical schools. This was the case with the first periodicals of New York, Philadelphia, Baltimore, Boston, Lexington, and I know not how many other places. Of all the journals of the West and South, those originally started here, and the two in New Orleans are, I believe, the only ones, not put forth directly or indirectly by those engaged in teaching. Here then we have the main cause of that aspect of our periodical literature, which must strike our transatlantic brethren as very remarkable.

Having suggested the cause of this unparalleled growth of medical journalism in our country, he is naturally led to speak of its effects. His remarks on this point will strike every one as sagacious and just. They are as follows:—

One of the most obvious benefits is an increase of the number of readers. Many physicians subscribe for a journal published near to them, who would not send to a great distance; others through feelings of State pride, and others still from its being conducted by the professors of their alma mater. And, although these motives are not of a high order, the acts to which they prompt are productive of improvements. Another benefit resulting from the multiplication of journals and their wide dissemination over the country is, that they call forth communications from neighboring physicians, who would never be aroused or emboldened to write for distant and perhaps more distinguished periodicals; and thus our science receives many contributions of facts, which otherwise would perish with those who observed them, while the writers themselves are by the very efforts stimulated to improvement. On the other hand, it is undeniable, that the demand for new materials is so far beyond the supply that much finds its way to our periodical press which, by its want of originality or intrinsic importance, with a still greater lack of simplicity and purity of style, retards the improvement of our medical literature, and postpones the time when it will enjoy the confidence and receive the homage of men of profound science or accomplished scholarship. Still further, the want of extended and productive subscription lists, renders it impossible for pubishers to make such compensation for communications as would greatly contribute not only to swell their number, but improve their quality. In every country, as far as I can learn, not less than in our own, physicians, as a class, are either poor, or in moderate pecuniary circumstances. The exceptions are chiefly found in the large cities of the world, and even there, to speak in our good and strong mother tongue, the majority barely contrive to make a living. It can not be doubted, then, that a liberal compensation to writers would act as a most effective stimulant, and greatly promote the elevation of our profession. The time when this desirable state of things will exist is, I fear, remote. The causes which continue to multiply our journals, must first exhaust themselves. Meanwhile new ones will be freqently starting up. It is but a few weeks since a physician, residing in a State where three journals now exist, did me the honor to ask my opinion about his establishing a fourth. I felt bound to advise him against it.

But notwithstanding the great number already in existence, Dr. Drake thinks there is still *one* more wanting in the United States, and, again to use his own language—

That should not be local, but continental; not analytical and analectic, but critical; not a vehicle for communications, but of reviews; not in the interest of any *one* of our thirty odd schools, but of the whole; not suborned to the business of those who republish foreign books, but fitted to teach them that all re-publications should be works of sterling merit; finally, not disposed to enquire what Europe has said of a book before speaking out, but resolved to make Europe ask, what does America say of it? Such a review, which might give whatever is new and valuable in all the important works of the day, with due condemnation of the remainder, is, at this moment, a *desideratum.* It would become at once a center of attraction and radiation, like the sun which keeps the planets in their orbits, while his rays impart life and joy to all who inhabit them. It might well be asked, who among us can do these things? The question is too hard for me to answer.

We have copied largely from this discourse, because we believed the topics were such as possessed a general interest, and were handled with ability. The author is a veteran editor as well as teacher, and is thoroughly versed in all the matters pertaining to medical journalism. He has a right to speak on the subject, and, speaking as he does from an experience both ample and diversified, his opinions will be felt. It was our purpose, when we commenced this notice, to add some thoughts of our own on the state of our medical periodical literature; but Dr. Drake seems to us to have touched upon every point inviting observation, and what we should say would be but an amplification of his remarks.

A few pages at the conclusion of his second discourse are devoted to the advantages of a public library of choice books, such as are not ordinarily to be found in the libraries of our physicians. He shows that practitioners, for the most part, purchase the same books, the shelves of one exhibiting but a repetition of others, and that "with *such* resources only, improvement by reading is soon at an end." And he continues—

The true and healthful bibliothical element of the mind, consists of monographs, reports of original observations, and the systematic works, which, in different ages, have presented the existing facts and principles of the profession, as they appeared to men of genius and experience. Such works have been, at all times, appearing and disappearing. They are not like ordinary compilations superseded by others of a later imprint, but a new generation overlooks them. They are like great men who have retired from the public gaze. They lie embalmed in the truths with which they are penetrated and can not decay; but like the bodies of kings and philosophers, steeped in spices and deposited in the catacombs, they are found only in unfrequented closets and alcoves. Now and then some of them are brought to the light and resuscitated, as has lately been done by the Sydenham Society; but such editions are soon exhausted and they again retire from the eye of the ordinary practitioner. It is to this class of works that our Association will, I hope, turn its attention.

Speaking of the libraries of the Medical College of Ohio, Transylvania University, and the University of Louisville, he says, "they contain many valuable books, but neither of them is growing, and none is open to the public."

We copy this remark for the purpose of correcting an error into which it seems strange that Dr. Drake should have fallen. Whether the libraries of the Medical College of Ohio and Transylvania University are growing or not, we have no certain information; but that additions are made every year to the library of the University of Louisville is certainly true. Many valuable additions to it have been made recently, and further additions will be made in the course of the present season. It is the determination of the Trustees and Faculty that it shall not stand still. Further than this—"it *is* open to the public," precisely in the sense in which any library in our country is open. Any physician in Louisville may obtain access to it by paying an annual fee of five dollars, and we suppose the members of the "Library Association of Cincinnati," will hardly get off with less. So, we repeat, the medical library of the University of Louisville is both a growing and an accessible library. It might have grown faster than it has done; it will grow faster in future, and all the more rapidly for the generous rivalry of our brethren at Cincinnati.

The last of these discourses was written before the first, and was evidently elaborated with great care. Both possess the charm of freshness, and cannot fail to be extensively read.

